# An Electronic Health Record–Based Automated Self-Rescheduling Tool to Improve Patient Access: Retrospective Cohort Study

**DOI:** 10.2196/52071

**Published:** 2024-03-19

**Authors:** Smitha Ganeshan, Andrew W Liu, Anne Kroeger, Prerna Anand, Richard Seefeldt, Alexis Regner, Diana Vaughn, Anobel Y Odisho, Michelle Mourad

**Affiliations:** 1 Department of Medicine University of California San Francisco San Francisco, CA United States; 2 Department of Urology University of California San Francisco San Francisco, CA United States; 3 UCSF Health Faculty Practices University of California San Francisco San Francisco, CA United States

**Keywords:** appointment, consultation, cost, digital health, digital tools, electronic health record, EHR, informatics, patient access, retrospective review, revenue, self-rescheduling tool, self-scheduling, waiting time

## Abstract

**Background:**

In many large health centers, patients face long appointment wait times and difficulties accessing care. Last-minute cancellations and patient no-shows leave unfilled slots in a clinician’s schedule, exacerbating delays in care from poor access. The mismatch between the supply of outpatient appointments and patient demand has led health systems to adopt many tools and strategies to minimize appointment no-show rates and fill open slots left by patient cancellations.

**Objective:**

We evaluated an electronic health record (EHR)–based self-scheduling tool, Fast Pass, at a large academic medical center to understand the impacts of the tool on the ability to fill cancelled appointment slots, patient access to earlier appointments, and clinical revenue from visits that may otherwise have gone unscheduled.

**Methods:**

In this retrospective cohort study, we extracted Fast Pass appointment offers and scheduling data, including patient demographics, from the EHR between June 18, 2022, and March 9, 2023. We analyzed the outcomes of Fast Pass offers (accepted, declined, expired, and unavailable) and the outcomes of scheduled appointments resulting from accepted Fast Pass offers (completed, canceled, and no-show). We stratified outcomes based on appointment specialty. For each specialty, the patient service revenue from appointments filled by Fast Pass was calculated using the visit slots filled, the payer mix of the appointments, and the contribution margin by payer.

**Results:**

From June 18 to March 9, 2023, there were a total of 60,660 Fast Pass offers sent to patients for 21,978 available appointments. Of these offers, 6603 (11%) were accepted across all departments, and 5399 (8.9%) visits were completed. Patients were seen a median (IQR) of 14 (4-33) days sooner for their appointments. In a multivariate logistic regression model with primary outcome Fast Pass offer acceptance, patients who were aged 65 years or older (vs 20-40 years; *P*=.005 odds ratio [OR] 0.86, 95% CI 0.78-0.96), other ethnicity (vs White; *P*<.001, OR 0.84, 95% CI 0.77-0.91), primarily Chinese speakers (*P*<.001; OR 0.62, 95% CI 0.49-0.79), and other language speakers (vs English speakers; *P*=.001; OR 0.71, 95% CI 0.57-0.87) were less likely to accept an offer. Fast Pass added 2576 patient service hours to the clinical schedule, with a median (IQR) of 251 (216-322) hours per month. The estimated value of physician fees from these visits scheduled through 9 months of Fast Pass scheduling in professional fees at our institution was US $3 million.

**Conclusions:**

Self-scheduling tools that provide patients with an opportunity to schedule into cancelled or unfilled appointment slots have the potential to improve patient access and efficiently capture additional revenue from filling unfilled slots. The demographics of the patients accepting these offers suggest that such digital tools may exacerbate inequities in access.

## Introduction

Health care access continues to be a concern as patients endure long wait times to access care [1,2]. In an industry survey, more than half of hospital leaders said that it takes more than 2 weeks to schedule patients on average and that access to specialty care is worse than before the COVID-19 pandemic [3]. Many health systems lack an efficient process to manage unfilled slots from cancellations. Unfilled cancellations and no-shows may exacerbate patient access issues. The process of scheduling and rescheduling appointments often requires significant time and manual labor, as staff members spend time calling patients to schedule, confirm, and reschedule appointments [4].

At busy medical centers, the high volumes of patient visits make this manual process of maintaining waitlists and filling cancelled appointments impractical. More recently, health care systems have used a range of tools to improve appointment completion rates and the process of scheduling patients into unfilled slots. Electronic health record (EHR)–based scheduling tools, phone call reminders, and automated SMS text message appointment reminders can all help ensure patients select an appointment time that works with their schedule, have the option to reschedule, and are prompted to confirm their appointment. EHR-based scheduling tools that allow patients to view available appointments as well as schedule, cancel, and reschedule their appointments through an app or a web-based portal saw a dramatic rise in use during the COVID-19 pandemic, with studies showing improved patient satisfaction [5-10]. EHR self-scheduling tools have been deployed in a variety of use cases, including the COVID-19 vaccine, radiology imaging, and well-child primary care visit scheduling [10,11]. Tools that allow patients to view and schedule themselves into available appointments may allow earlier and easier access for patients, reduce staff burden, and increase clinic volume. More advanced self-scheduling tools can allow for the maintenance of an electronic waitlist that can provide patients with notifications when an earlier appointment is available. Despite these reported benefits, data quantifying the efficacy of these tools, their impact on patient access, and their financial value for health systems are needed to help spur adoption and investment [12].

The University of California, San Francisco (UCSF) Medical Center implemented an EHR module, Fast Pass (Epic Systems Corporation). The Fast Pass module, which is available through Epic’s suite of self-scheduling tools, allows a patient to add themselves to a waitlist for an earlier appointment slot. When a slot becomes available, patients receive an automated notification through SMS text message or email prompting them to log into their patient portal (MyChart) to self-schedule into the new, earlier appointment slot or to keep their existing appointment slot. As an existing module within the Epic EHR, included as part of the MyChart patient portal, there was limited integration necessary, as would be required with a third-party vendor. While the Fast Pass tool had previously been available through Epic, there had been no enterprise-wide implementation effort at UCSF to facilitate its use.

The primary objective of this study was to describe the uptake of the enterprise-wide Fast Pass scheduling tool among patients, understand the impacts of Fast Pass on patient wait times for appointments, and determine the potential incremental dollar value of visits that may have otherwise remained unscheduled. Our secondary aim was to study the uptake of the tool by specialty, given the unique patient needs and workflows in each specialty.

## Methods

### Setting

UCSF Health is a large academic health system with 3 campuses, over 1000 inpatient beds, and 9 primary care practices serving approximately 90,000 patients. UCSF Health has approximately 45,000 hospital admissions and 1.7 million outpatient visits annually. As of January 2022, approximately 89% of adult ambulatory care patients were enrolled in UCSF’s EHR-tethered patient portal. There are a total of 1538 different department entities across all service areas at UCSF. Before November 2022, 103 different departments were sending offers through Fast Pass, and by January 2023, the number of departments sending Fast Pass offers had increased to 220.

### Fast Pass Implementation at UCSF

To use Fast Pass, a patient opts into the program through the patient portal and elects to receive notifications through email, SMS text messaging, or both regarding earlier appointment slots as they become available. Fast Pass offers are sent in batches in the evening to multiple patients for a single appointment slot, and patients have 12 hours to sign up for the earlier slot, after which the offer expires. When patients log into their patient portal, they can self-schedule into or decline the earlier appointment. When a patient declines, they have the opportunity to receive another offer at a different time. Beginning in November 2022, a central multidisciplinary team was formed to implement the Fast Pass tool across all departments at UCSF. Staff at the clinics were trained on using and implementing this tool in their respective clinics. A tip sheet was developed to facilitate patient education and awareness, and the tool was added to the UCSF digital and website promotions.

### Data Acquisition

Fast Pass offer data from June 18, 2022, to March 9, 2023, for the 220 departments included in the Fast Pass implementation were extracted from the EHR. The demographic and scheduling data associated with the Fast Pass offers were included in the data extraction. Demographic data included patient age, gender, race and ethnicity, insurance financial class, marital status, and primary language. Fast Pass data included the offering department, provider name, visit type, whether the patient had an existing appointment, offer sent date and time, offered slot date and time, visit length, and the date and time the offer was first viewed. Visit types were stratified by in-person office visits (including radiology and procedures), video visits, and nonbillable phone calls. Nonbillable phone calls were excluded from the analysis. Clinics offering the Fast Pass feature were grouped into their respective specialties by a physician-informaticist familiar with the UCSF system and EHR department entities.

The primary exposure of our retrospective cohort study was the dichotomous variable of whether the clinic had access to the enterprise Fast Pass tool. We only analyzed results from clinics that had access to Fast Pass in this study. Fast Pass appointment offers were also stratified according to their 4 possible categorical outcomes: accepted, declined, expired, or unavailable (offer accepted by another patient first). Additionally, Fast Pass–accepted appointments were stratified by 3 possible categorical appointment outcome end points: completed, no-show, and cancelled.

### Data Analysis

Descriptive statistics were performed. We compared differences in the patient cohort stratified by Fast Pass offer acceptance status (accepted vs nonaccepted) using the chi-square test for categorical features. A multivariate logistic regression model was built with primary outcome of offer acceptance status to determine predictors of offer acceptance and the size of associations between primary outcome and the demographic factors extracted from the EHR as additional independent variables chosen by theoretical criteria. No stepwise regression was conducted. Offer acceptance status was used as the primary outcome due to Fast Pass functionality primarily being designed to increase the number of appointment slots filled rather than decrease the number of no-shows or cancellations. All analysis was conducted in R (version 3.5.1; R Foundation for Statistical Computing), and a value of *P*<.05 was considered significant.

### Analysis of Fast Pass by Departments

Descriptive analyses were conducted on the outcomes of offers as well as the outcomes of accepted appointments, stratified by specialty.

### Visit Dollar Value Calculation

We extracted the Fast Pass offers that were accepted and completed in a 9-month period and calculated the dollar value of the professional fees for these visits. We used the number of visits that Fast Pass added to the schedule within 7 days of the appointment in the 9 months of implementation, the payer mixes for these visits, and the average professional fee collections for the visits by specialty and by payer to estimate the revenue of the added visits. We hypothesize that visits scheduled within 7-days of an appointment were more likely to remain unfilled. These revenue numbers only include physician evaluation and management revenue, not facility or technical fees. We do not include downstream revenue associated with patient visits. While these Fast Pass visits do not represent true net new patient service revenue, they help fill a proportion of cancelled slots that may otherwise have gone unfilled and may create downstream capacity for new patients.

### Ethical Considerations

This study was reviewed and approved by the UCSF institutional review board (22–35948) and was determined to not be human participants research. Epic Systems did not fund this study or participate in the analysis, and it is a paid vendor of UCSF.

## Results

From June 18, 2022, to March 9, 2023, there were a total of 60,660 Fast Pass offers for 21,978 appointments sent to patients. As of March 17, 2023, there were 182 offers currently active or deleted on the clinic’s end, for a total of 60,478 offers analyzed. The median (IQR) number of requests sent for each appointment was 2 (1-3). Out of 60,478 offers, there were 6703 (11%) offers accepted, for a monthly median of 139 visits across all departments. Of the 21,978 appointments sent, 6703 offers were accepted, for a 30.5% success rate. Of the accepted offers, 294 were beyond the time period of this analysis and therefore excluded, resulting in 6409 total analyzed accepted offers. Of the accepted offers within the time period of this study, 5399 appointments were completed, resulting in an overall completed appointment from the Fast Pass offer rate of 84.2%.

The cohort of patients who accepted their offer was significantly more likely to be older, male, White, have English as their primary language, and have an in-person visit, a physician visit, and a shorter appointment as part of their Fast Pass offer (Table 1). There was no difference in Fast Pass offer acceptance by insurance class. In addition, among patients that accepted a Fast Pass offer, patients who were older, male, of White race or ethnicity, had commercial insurance, saw a physician provider, and had a shorter appointment time were more likely to complete their appointment. There were no differences in appointment completion rates between visit types or primary languages.

**Figure 1:**
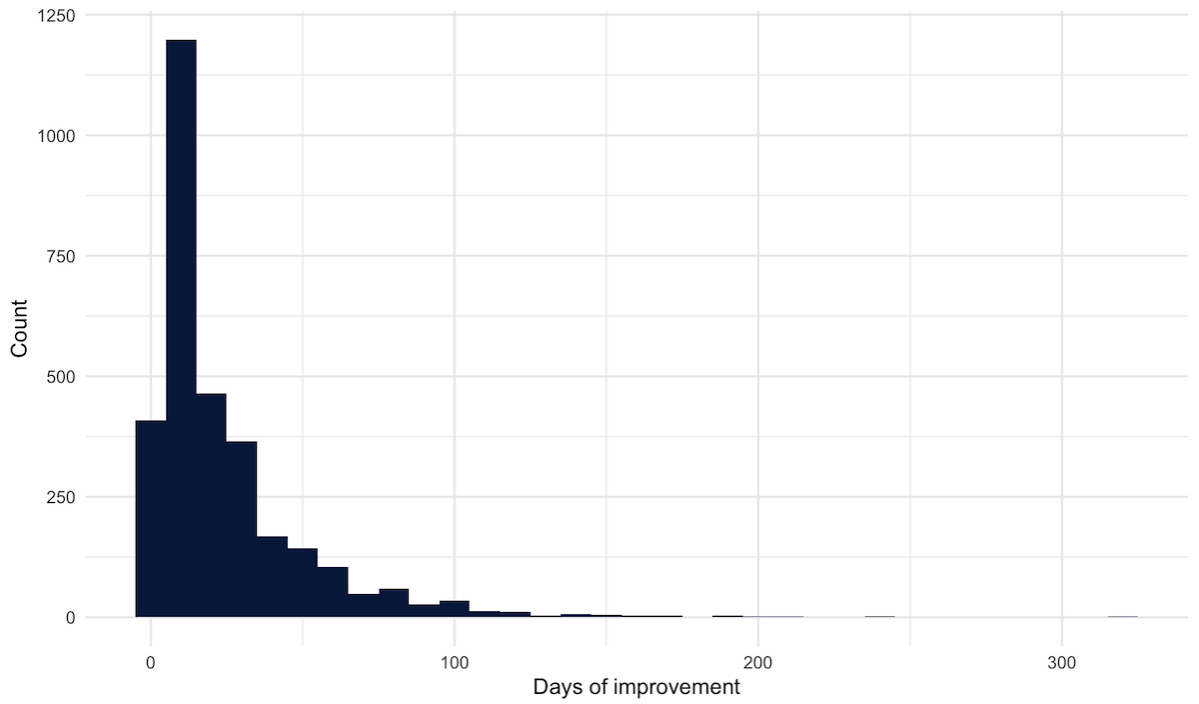
Distribution of number of days improvement from existing appointment and rescheduled appointment through Fast Pass.

In a multivariate regression model with the primary outcome of Fast Pass appointment offer acceptance, patients who were aged 65 years or older (odds ratio [OR] 0.86, 95% CI 0.78-0.96) versus patients who were aged younger than 40 years, other ethnicity (OR 0.84, 95% CI 0.77-0.91) versus White patients, primarily Chinese speakers (OR 0.62, 95% CI 0.49-0.79) and other language speakers (OR 0.71, 95% CI 0.57-0.87) versus English-speaking patients were less likely to accept an offer (Table 2). Male patients (OR 1.09, 95% CI 1.03-1.15) were more likely to accept their offer compared with female patients. In terms of the Fast Pass offer details, compared to office visits, phone calls were 41% less likely to be accepted (OR 0.59, 95% CI 0.38-0.86), while video visits were 29% more likely to be accepted (OR 1.29, 95% CI 1.20-1.38). A Fast Pass offer with a physician was 55% more likely to be accepted (OR 1.55, 95% CI 1.46-1.63) compared with a nonphysician offer, while appointments longer than 45 minutes (OR 1.20, 95% CI 1.09-1.32) and between 20 minutes and 45 minutes (OR 1.15, 95% CI 1.09-1.22) were both more likely to be accepted versus those less than 20 minutes. Patients were seen a median (IQR) of 14 (7-33) days before their originally scheduled appointment for those that eventually completed their visits from a Fast Pass offer (Figure 1).

**Table 1:** Demographics of Fast Pass offers stratified by offer acceptance.

		Accepted (n=6703), n (%)		Not accepted (n=53,775), n (%)			*P* value	
**Patient age (years)**								.01
	18-40	1637 (24.4)	12,307 (22.9)					
	40-64	2839 (42.4)	23,033 (42.8)					
	≥65	2227 (33.2)	18,435 (34.3)					
**Gender**								.004
	Male	2585 (38.6)	19,649 (36.6)					
	Female	4038 (60.2)	33,418 (62.1)					
	Other or unknown	80 (1.2)	708 (1.3)					
**Ethnicity**								<.001
	White	3807 (56.8)	28,825 (53.6)					
	Black or African American	318 (4.7)	2717 (5.1)					
	Hispanic or Latino	649 (9.7)	5102 (9.5)					
	Asian, Native Hawaiian, or other Pacific Islander	1137 (17.0)	99,76 (18.6)					
	Other or unknown	792 (11.8)	7155 (13.3)					
**Primary language**								<.001
	English	6462 (96.4)	50,832 (94.5)					
	Spanish	68 (1.0)	677 (1.3)					
	Chinese	76 (1.1)	1110 (2.1)					
	Other	97 (1.4)	1156 (2.1)					
**Insurance**								.43
	Commercial	3805 (56.9)	30,217 (56.3)					
	Medicare	703 (29.8)	5715 (30.0)					
	Medicaid	1994 (10.5)	16,099 (10.6)					
	Other	186 (2.8)	1676 (3.1)					
**Provider type**								<.001
	Physician	3994 (59.6)	27,933 (48.1)					
	Nonphysician	2709 (40.4)	25,842 (41.9)					
**Offered visit type**								<.001
	Office visit, radiology, or procedure	5212 (77.9)	45,109 (84.0)					
	Video visit	1465 (21.9)	291 (0.5)					
	Nonbillable phone call	26 (0.4)	8375 (15.6)					
**Offered appointment length (minutes)**								<.001
	<20	2813 (42.1)			24,580 (45.8)			
	20-45	3183 (47.5)			24,215 (45.0)			
	>45	700 (10.4)			4965 (9.2)			

**Table 2:** Multivariate predictive model for Fast Pass use.

Term			OR^a^	95% CI	*P* value
**Patient age (vs <40 years)**					
	40-64		0.96	0.89-1.01	.11
	≥65		0.86	0.78-0.96	.005
**Sex (vs female)**					
	Male		1.09	1.03-1.15	.002
	Other or unknown		0.91	0.71-1.15	.45
**Ethnicity (vs White)**					
	Black or African American		0.92	0.81-1.04	.19
	Hispanic or Latino		1.03	0.94-1.14	.49
	Asian, Native Hawaiian, or other Pacific Islander		0.97	0.90-1.04	.39
	Other or unknown		0.84	0.77-0.91	<.001
**Primary Language (vs English)**					
	Spanish		0.79	0.60-1.02	.09
	Chinese		0.62	0.49-0.79	<.001
	Other		0.71	0.57-0.87	.001
**Insurance (vs commercial)**					
	Medicare		1.06	0.97-1.16	.21
	Medicaid		1.04	0.94-1.14	.46
	Other		0.93	0.79-1.08	.36
**Visit type (vs office visit)**					
	Nonbillable phone call		0.59	0.38-0.86	<.001
	Video visit		1.29	1.20-1.38	<.001
**Provider type (vs nonphysician)**					
	Physician		1.55	1.46-1.63	<.001
**Appointment length (minutes)**					
	20-45		1.15	1.09-1.22	<.001
	>45		1.20	1.09-1.32	<.001

^a^OR: odds ratio.

In a multivariate regression model with primary outcome Fast Pass appointment outcome post–offer acceptance, older patients (aged 40-64 years: OR 1.32, 95% CI 1.11-1.56; *P*=.001; and aged 65 years or older: OR 1.68, 95% CI 1.27-2.22; *P*<.001) versus patients aged 40 years or younger, and male patients (OR 1.35, 95% CI 1.17-1.57; *P*<.001) were more likely to complete their appointment. Patients with Medicaid (OR 0.51, 95% CI 0.42-0.63; *P*<.001), Medicare (OR 0.64, 95% CI 0.50-0.84; *P*<.001), and other insurance (OR 0.47, 95% CI 0.33-0.68; *P*<.001) versus commercial, and patients with appointment lengths longer than 45 minutes (OR 0.79, 95% CI 0.63-1.00; *P*=.05) compared with appointments less than 20 minutes in length were less likely to complete their appointment. There were no observed differences in the patient cohort in terms of race or ethnicity, primary language, visit type, or provider type for appointment completion.

**Figure 2:**
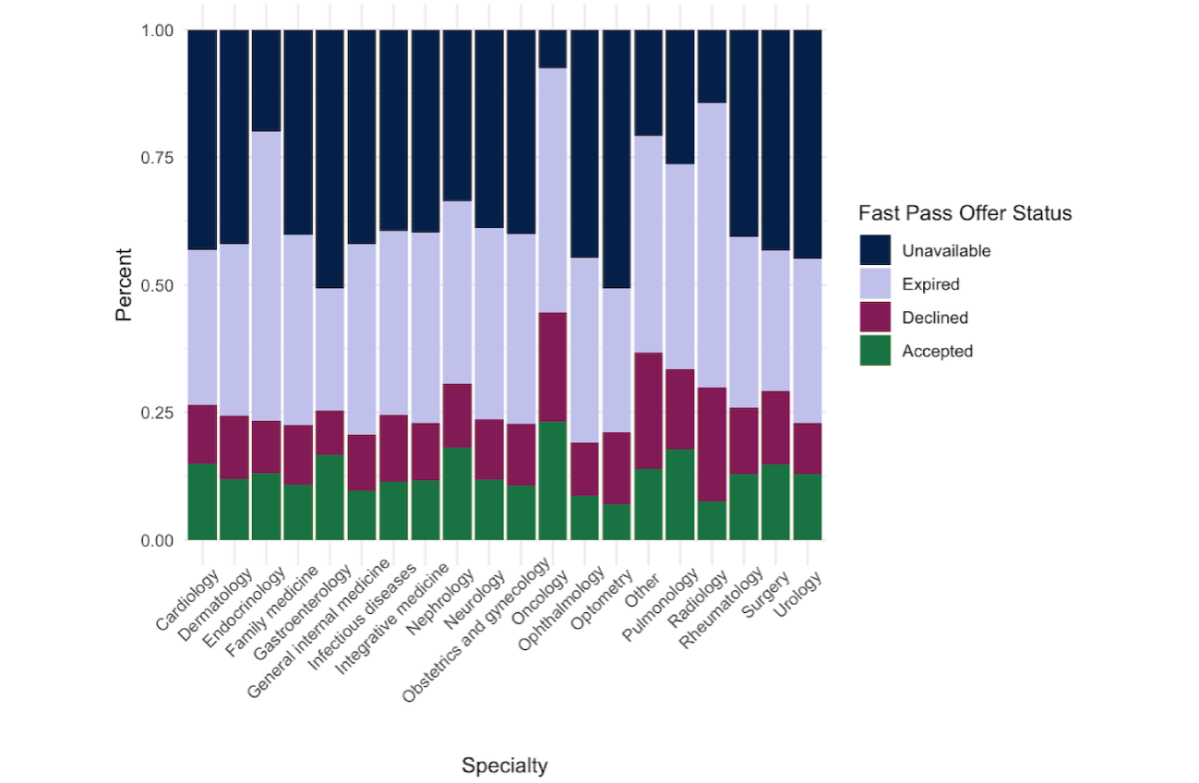
Outcomes of Fast Pass offers: unavailable, expired, declined, and accepted.

**Figure 3:**
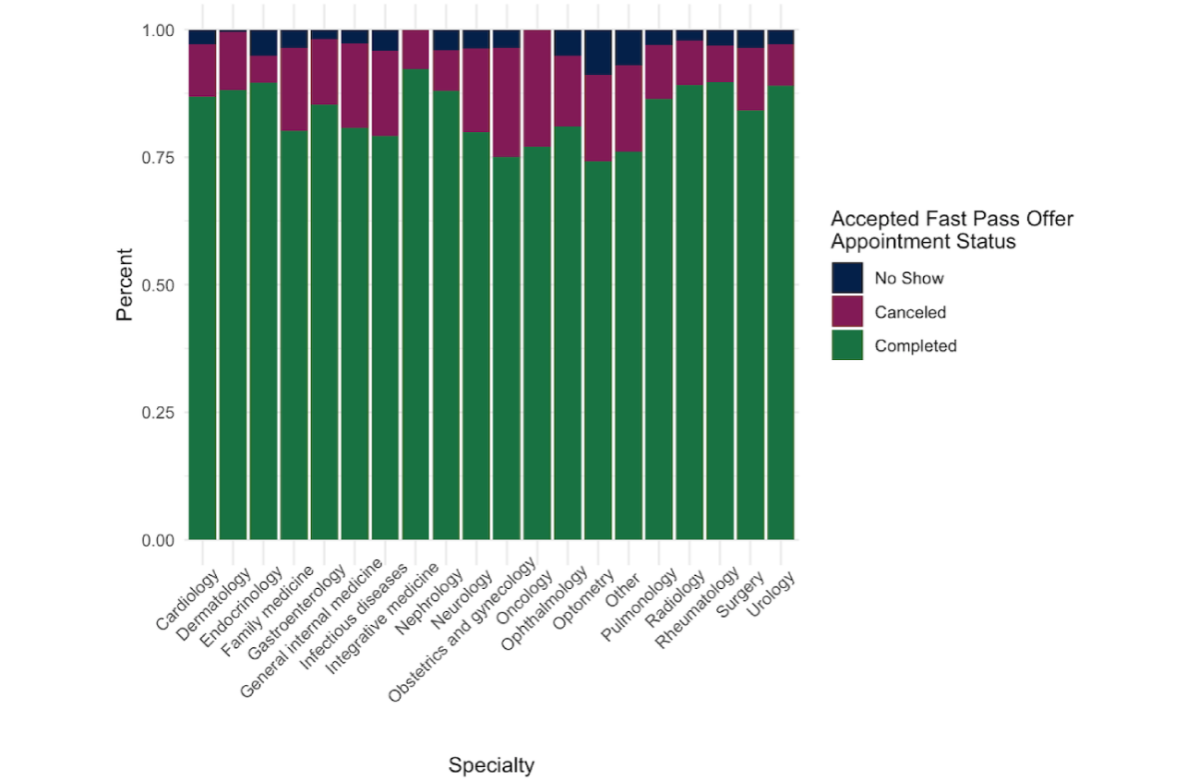
Outcomes of accepted Fast Pass offers: no show, canceled, and completed.

There was differential uptake of Fast Pass among the specialties at our institution, both by offers accepted and by rates of completed appointments from Fast Pass offers (Figure 2). Oncology (49/211, 23.2%), nephrology (78/431, 18.1%), and pulmonology (70/394, 17.8%) were the specialties with the highest rates of fast pass scheduling offers accepted, while optometry (127/1831, 6.9%), radiology (1459/19134, 7.6%), and integrative medicine (114/283, 8.9%) had the lowest percentage of accepted fast pass offers. Rheumatology (139/155, 89.6%), endocrinology (69/77, 89.6%), and nephrology (66/75, 88.0%) had the highest rates of completed appointments using fast pass offers, while optometry (92/124, 74.1%), obstetrics and gynecology (314/429, 73.2%), and infectious diseases (31/42, 73.8%) had the lowest percentage (Figure 3).

In the Fast Pass revenue analysis, a total of 5387 of the 5399 completed visits added in the first 9 months of implementation across 25 specialties had payer information available and were included in the analysis (Table 3). These visits represent an estimated US $3 million in professional fees.

**Table 3:** Dollar value of physician fees from Fast Pass visits.

Specialty	Commercial, n	Medicare, n	Medicaid, n	Other, n	Total, N	Total revenue (US $)
Allergy and immunology	14	5	1	0	20	7819.00
Cardiology	126	187	15	1	329	103,446.66
Dermatology	183	21	4	1	209	72,043.80
Endocrinology	41	15	10	2	68	25,550.09
Family medicine	66	33	10	10	119	29,710.25
Gastroenterology	93	51	25	6	175	67,427.70
General internal medicine	326	120	28	11	485	130,770.54
Hematology	4	4	0	0	8	2760.16
Infectious diseases	11	17	3	0	31	9159.47
Integrative medicine	40	29	8	2	79	24,713.67
Nephrology	19	42	5	0	66	16,908.58
Neurology	76	81	13	4	174	53,757.85
Obstetrics and gynecology	244	29	29	11	313	113,294.91
Occupational health	1	0	0	0	1	275.89
Oncology	23	10	2	2	37	14,267.10
Ophthalmology	48	58	13	5	124	26,609.56
Optometry	37	32	16	6	91	20,016.01
Other	121	63	22	9	215	67,280.05
Otolaryngology	16	6	0	0	22	7907.58
Palliative care	2	4	1	0	7	1848.01
Pulmonology	22	27	7	1	57	17,875.52
Radiology	819	240	177	36	1272	1656,417.00
Rheumatology	82	40	17	0	139	49,700.57
Surgery	563	340	73	20	996	308,187.04
Transplant	1	2	1	0	4	1079.55
Urology	159	149	31	7	346	103,267.57
Total	3137	1605	511	134	5387	2,932,094

## Discussion

### Overview

FFast Pass is an EHR-based tool that allows patients to schedule into earlier appointment slots and allows clinics to fill unfilled appointments without the significant manual staff time needed to call and reschedule appointments in a timely manner. This study found that patients saw a median improvement in their appointment time slot between existing and rescheduled appointments of 14 days, which supports the ability of self-scheduling tools to facilitate quicker patient access to care. From June 18, 2022, to March 9, 2023, there were a total of 60,660 Fast Pass offers sent to patients. Of these offers, 6703 (11%) Fast Pass offers were accepted across all departments, for a monthly median of 139 visits across all departments. The median (IQR) number of requests for each appointment slot was 2 (1-2), which further highlights that this tool can free up staff time for direct patient care tasks or to assist patients who are less familiar with the EHR patient portal. This study builds on existing literature on the Fast Pass EHR tool by supporting its feasibility and implementation in a large tertiary health system and adding additional data on patient use of the tool, impacts across specialties, and potential revenue impacts [12].

When we analyzed Fast Pass offers by specialty, we found differential uptake of Fast Pass among the specialties at our institution. Oncology, nephrology, and pulmonology were the specialties with the highest rates of fast pass scheduling offers, while optometry, radiology, and integrative medicine had the lowest percentage of accepted fast pass offers. It is possible that our oncology, nephrology, and pulmonology clinics have longer wait times for ambulatory visits or higher cancellation rates, and therefore, the clinics and patients gain more value from automated Fast Pass offers and scheduling. Additional qualitative research may help us understand the drivers of differential uptake to better facilitate enterprise-wide implementation efforts of scheduling tools.

In our analysis, Fast Pass added 2576 patient service hours to the clinical schedule, with a median (IQR) of 251 (216-322) hours per month. The dollar value of physician fees from these visits through Fast Pass scheduling for 9 months of Fast Pass at our institution was approximately US $3 million. While this dollar value does not necessarily equate to the net additional revenue impact of Fast Pass, it does help quantify the financial impacts of self-scheduling tools since Fast Pass is an add-on tool in the EHR with minimal incremental cost [4]. Collecting additional longitudinal data will help us better understand the overall impacts of Fast Pass on ambulatory visit volumes, template use, patient access, and no-show rates.

This study builds on existing demographic data on digital tools, which show that patients who are White, in a younger age group, and have commercial insurance are more likely to use the tool [11,13-17]. These data raise concerns about equity in access to digital tools as health systems increasingly rely on EHR applications to facilitate front- and back-office functions, including triage and scheduling. Our data suggests that Fast Pass and similar tools may limit access to earlier appointments for older, non-White patients with a preferred language other than English. Given the personnel cost savings, these digital tools are likely to increase in use, and resources need to be channeled into supporting equitable adoption by all patients, or in the case of scheduling, ensuring that a system is put in place for offering patients earlier appointments based on clinical urgency. Possible interventions that may be undertaken to improve health equity in the area of self-scheduling include outreach of the tool in multiple languages or product design to increase accessibility for patients with low technological literacy.

### Limitations

Our study has several limitations. This experience at an urban academic medical center with a wide clinical catchment area across California may not be generalizable to other regions of the country or other institutions, especially facilities with fewer subspecialty referrals or with different EHRs lacking a scheduling tool directly connected with their patient portal. This was also designed as a retrospective study. Future prospective natural experiments that test different modes of patient and clinician engagement could identify the key factors necessary for the successful implementation of this program enterprise-wide. In addition, the use of Fast Pass was limited to patients who had access to their MyChart portal. Previous data have demonstrated inequities in the ability to access and use MyChart, which may further compound inequities in using the Fast Pass tool. We did not have data to access patient digital literacy, which may play an important role in Fast Pass use. Further qualitative data are needed to better understand the specific patient and clinical workflow factors that lead to differential use of Fast Pass across specialties and departments.

### Conclusions

Fast Pass, an EHR-based self-scheduling tool, afforded patients the opportunity for an earlier appointment and provided our medical center with the opportunity to efficiently capture revenue from cancelled appointments. We found that the patients accepting these offers were more likely to be older, male, and English-speaking, suggesting that these digital tools could exacerbate inequities in access.

## Abbreviations

EHR: electronic health record
UCSF: University of California, San Francisco
